# Early detection of *Coxiella burnetii* growth on axenic media using scanning electron microscopy

**DOI:** 10.1128/spectrum.01011-25

**Published:** 2025-12-23

**Authors:** Asmae El Moutawakil, Asmaa Elomrani, Omar Zmerli, Jacques Bou Khalil, Pierre-Edouard Fournier, Rita Abou Abdallah

**Affiliations:** 1 IHU-Méditerranée Infection 290815, Marseille, France; 2 Aix Marseille University, IRD, AP-HM, SSA, RITMES 128791, Marseille, France; 3 Aix Marseille University, MEPHI 683946 , Marseille, France; UJF-Grenoble 1, CHU Grenoble, Grenoble, France

**Keywords:** axenic medium, scanning electron microscopy, growth detection, *Coxiella burnetii*

## Abstract

*Coxiella burnetii*, the causative agent of Q fever, is a highly infectious obligate intracellular bacterium. In consequence, cultivating this pathogen was fastidious and resource-consuming, requiring 3 weeks for isolation in cell culture. In recent years, axenic media allowing *C. burnetii* growth without eukaryotic cells were developed. These media reduced the complexity of its manipulation. However, despite this advancement, traditional growth monitoring methods such as cell culture, colony counts, and immunofluorescence remain time-consuming, requiring an average of 10–14 days to detect growth. However, this is still considered too long in the context of research, epidemiologic surveillance, and treatment failure. Here, we present a novel method that coupled axenic growth to early detection using the TM4000Plus II-Tabletop scanning electron microscopy (SEM) (Hitachi, Japan). For this, we used eight strains of *C. burnetii* that were cultured in axenic medium and then prepared for SEM observation. Growth was assessed by the calculation of the percentage area coverage. We were able to detect growth within only 3 days with a percentage area coverage superior to 60%. This result was reproducible among the eight strains. Finally, the use of SEM for early growth detection of *C. burnetii* in axenic medium is simple and feasible and may reduce detection time by 70%. This promising approach may also provide deeper insights into the morphological details, metabolic, and growth kinetics of this intracellular bacterium.

**IMPORTANCE:**

*Coxiella burnetii* is the causative agent of Q fever, a serious disease affecting humans and other mammals. Q fever outbreaks are reported worldwide, posing significant public health and economic concerns. While cultivating this pathogen is not typically required for diagnosis, it remains valuable in cases involving new clinical presentation, treatment failure, or atypical epidemiological situations. This highlights the need for novel techniques that are both simple and capable of enabling early growth detection. Here, we present an innovative and simple approach allowing early growth detection of *C. burnetii* in axenic media using scanning electron microscopy.

## INTRODUCTION

*Coxiella burnetii* is the causative agent of a zoonotic disease named Q (query) fever (1). It is a highly virulent intracellular pathogen, known for its environmental resilience and zoonotic reservoir presenting serious risks to public health (2, 3). *C. burnetii* cells are a gram-negative-like pleomorphic bacilli measuring 0.2–2 µm. *C. burnetii* shows two different morphological forms: large-cell variants, which are metabolically active, and small-cell variants, which are environmentally resilient and infectious (4, 5). This bacterium is widely distributed, mostly through infected aerosols and has been associated with serious outbreaks in livestock-rearing areas (6). Once inside the host, *C. burnetii* demonstrates a unique tropism for macrophages and monocytes (7). Clinically, Q fever is polymorphic, and its symptoms range from silent acute infection to a severe chronic disease (8). Acute Q fever often goes unnoticed but can be presented either as isolated fever or as a distinct form of pneumonia. Therefore, people with persisting valvular or vascular deficits may develop chronic Q fever in the form of endocarditis or vascular infection with a mortality of up to 25% (9). Regarding the treatment, doxycycline is the first-line antibiotic, forming the basis of therapeutic strategies (9). The current diagnostic approaches for Q fever include serological tests. However, molecular methods such as PCR are now widely used as the primary approach for routine diagnosis of *C. burnetii* infection due to their rapidity. However, their performance variation across settings and false-negative results remains a recognized issue. In contrast, cell culture remains the gold standard for confirmation but is rarely performed due to its complexity, biosafety requirements, and long time to result. Nevertheless, culture remains an important reference tool in specialized laboratories, where it enables confirmatory testing and further epidemiological or genomic characterization of isolates (10, 11). Historically, *C. burnetii* could only be cultivated in embryonated eggs (12) or eukaryotic cell cultures (13). Most commonly, *C. burnetii* is cultivated in mammalian cell lines such as Vero cells, derived from African green monkey kidney cells, or L929 cells, derived from mouse fibroblasts (14). Therefore, cultivating this pathogen is time and resource-consuming, requiring 3 weeks for isolation in cell culture. In recent years, the development of acidified citrate cysteine medium (ACCM), later refined into ACCM-2, revolutionized *C. burnetii* culture by enabling its growth outside host cells (15). This axenic medium, thus reduced the complexity of its manipulation and provided pure cultures essential for studies on metabolism, genome sequence, and pathogenesis (15). However, despite this advancement, traditional growth detection methods such as culture colony counts and immunofluorescence remain time-consuming, requiring an average of 7–14 days to detect growth. In addition, detection is still largely operator-dependent, relying on subjective methods such as naked-eye observation and optical microscopy. Consequently, the sensitivity of these approaches may be insufficient, leading to a poor assessment of bacterial growth.

In response to the challenges of traditional growth detection methods, this study aimed to explore the combination of axenic media with scanning electron microscopy (SEM) as a rapid and effective method for detecting *C. burnetii* growth. The primary objective was to assess the utility and potential of the novel tabletop scanning electron microscope in detecting bacterial growth earlier than traditional methods, by developing optimized SEM protocols that allow the observation of *C. burnetii* in axenic media and reduce the time needed for growth detection in comparison with traditional approaches.

## MATERIALS AND METHODS

### Strain collection and culture

The eight *C. burnetii* strains used in this study are listed in Table 1. All strains were obtained from the Collection de Souches de l’Unité des Rickettsies (CSUR). Bacteria were cultured in a slightly modified axenic medium, ACCM-2, adjusted to pH 4.75 to mimic the acidified parasitophorous vacuole environment, and the cultures were incubated at 37°C in a 5% CO_2_.

**TABLE 1:** *C. burnetii* strains used in this study

Strain
*C. burnetii* Nine Mile
*C. burnetii* Henzerling
*C. burnetii* 336
*C. burnetii* 109
*C. burnetii* 346
*C. burnetii* 284
*C. burnetii* 252
*C. burnetii* 350

### Growth monitoring by SEM

Growth monitoring consisted of daily SEM observation for each culture. The kinetic interval consisted of daily screening of cultures going from day 0 to day 7. All procedures were carried out in strict accordance with established protocols within our Biological Safety Level 3 (BSL-3) laboratory.

#### Sample preparation

**Fig 1:**
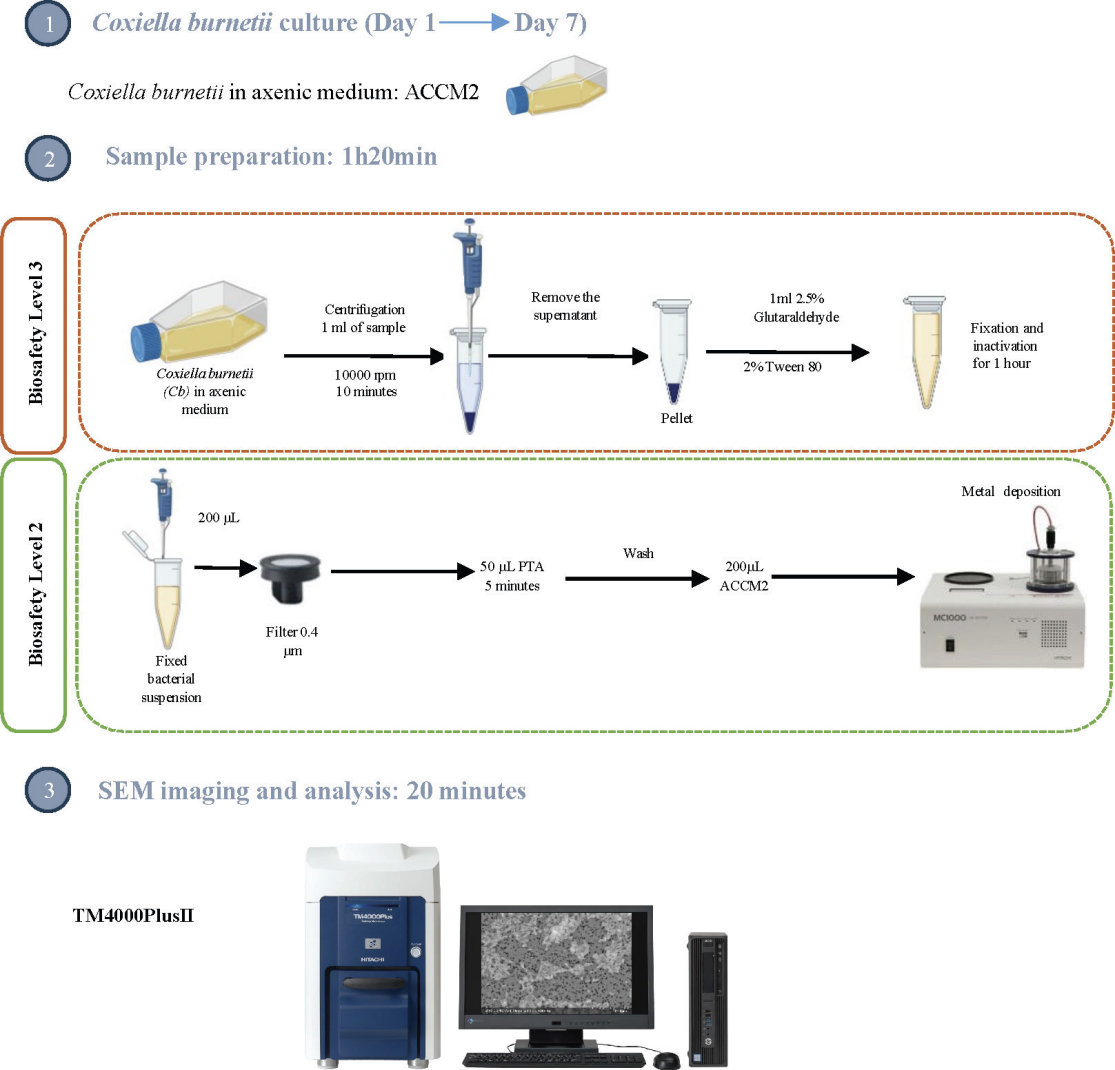
Detailed protocol of *C. burnetii* observation in ACCM-2 medium using scanning electron microscopy.

The sample preparation protocol was developed and optimized as a function of image quality and results reproducibility. First, 1 mL of *C. burnetii* culture was centrifuged to remove the supernatant. The bacterial pellet was then fixed and inactivated with 1 mL of 2.5% glutaraldehyde containing 2% Tween 80 for 1 h. Following inactivation, the bacterial suspension was securely transferred from the BSL-3 laboratory to the BSL-2 laboratory for further preparation and imaging. In the BSL-2 laboratory, 200 µL of the fixed bacteria were deposited onto 0.4 µm pore track etched polycarbonate filters (it4ip S.A, Belgium) mounted on nano-percolator sample mounts (Nisshin EM Co., Ltd, Japan) and excess liquid was removed by suction using a syringe. To enhance contrast for SEM visualization, the samples were stained with 50 µL of 10% phosphotungstic acid (PTA) (Sigma-Aldrich, St. Louis, MO, USA) for five minutes. After staining, excess PTA was removed by suction, and the filters were washed with 200 µL of ACCM-2 to eliminate any residual staining compounds. To enhance conductivity and reduce charging effects during SEM observation, the prepared samples were subjected to metal sputter coating using an MC1000 Ion Sputter Coater (Hitachi High-Tech, Japan). A thin layer of platinum-palladium alloy was then deposited onto the samples. This step was critical for preserving the fine morphological details of the bacteria and ensuring high-resolution imaging with optimal contrast and clarity during SEM analysis. Figure 1 shows the detailed sample preparation protocol.

#### SEM imaging

SEM imaging to observe daily *C. burnetii* growth over 7 days was performed using the TM4000 Plus II tabletopSEM (Hitachi High-Tech). Imaging was carried out under optimized conditions to ensure both sample stability and high-resolution visualization. Key parameters of TM4000 Plus included an accelerating voltage of 10 kV using Backscattered Electron (BSE) detector, which enhanced contrast and thus the detection of dense bacterial structures. To comprehensively evaluate bacterial growth, two magnification levels were used: low magnification (×1,000) for assessing the general distribution and surface coverage of bacteria on the filter, and high magnification (×3,000) to allow a close observation of bacterial morphology. The Zigzag imaging function for automated image acquisition was used, enabling the fast screening of the sample by randomly selecting several imaging zones that ensure complete representation of the filter, thus generating 150 images per condition. Acquisition settings appear on each micrograph in the following order: Instrument, Accelerating Voltage, Working Distance, Magnification, and Detector.

#### Image analysis

**Fig 2:**
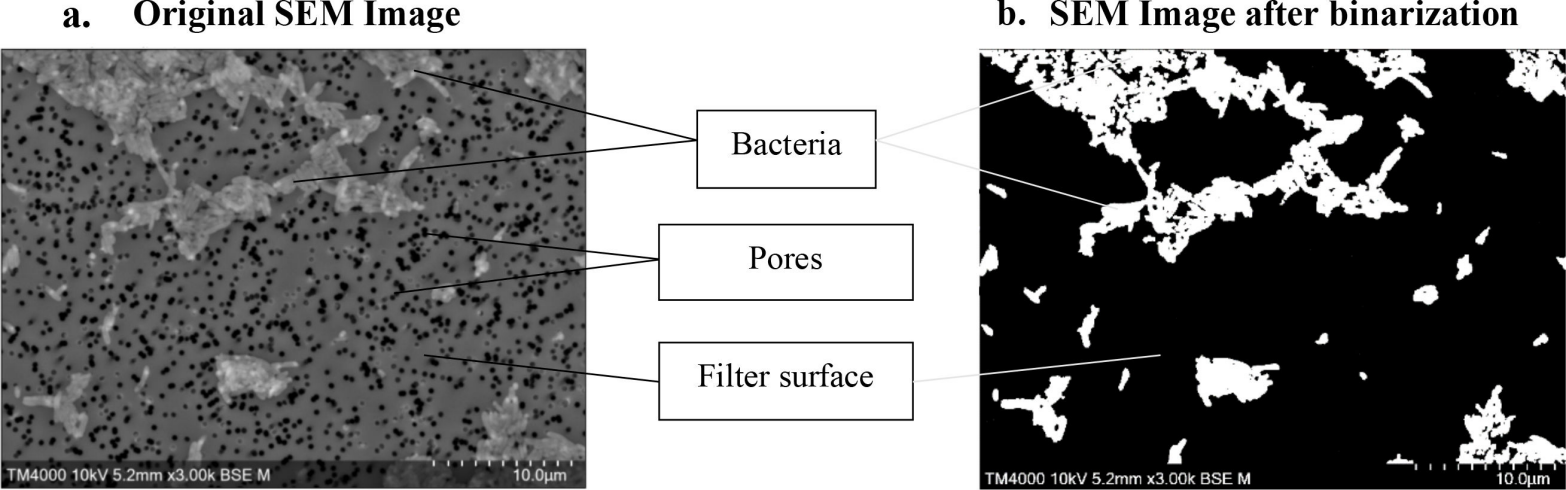
Image analysis using the Fiji ImageJ software. Example of *C. burnetii* strain 109. The same image is shown in Fig. 4 along with other analyzed conditions. (**a**) Original micrograph. (**b**) Binarized micrograph.

Generated micrographs were analyzed using Fiji ImageJ software (16), to calculate the surface area occupied by *C. burnetii*. Analysis was performed on 150 images. The results were expressed in terms of percentage, representing the surface of the filter that is covered by *C. burnetii* obtained from the mean of percentages of 150 images. Binarization by thresholding was performed on the SEM images to produce binary representations, with the surface covered by bacteria rendered in white against a black background free of bacteria. An empty filter subjected to the same preparation conditions without bacteria was used as a reference/blank. The growth evaluation over time was indirectly estimated by calculating the percentage area coverage; in other terms, a higher percentage area coverage indicates bacterial growth. This quantitative approach allowed us to monitor bacterial growth kinetics by tracking changes in surface area coverage across the experimental timeline. Figure 2 shows an example of the analysis protocol executed using Fiji ImageJ software on a SEM image for one of the chosen *C. burnetii* strains (Strain 109), which is the same image shown in Fig. 4 along with other conditions.

#### Statistical analysis

The Kruskal-Wallis test was used to compare the percentage area coverage across each day for the eight strains. The aim of this test was to assess the reproducibility of this method and to validate the time point for detection.

#### Quantitative detection with real-time PCR of *C. burnetii*

Deactivation was performed by incubating the bacterial suspension at 96°C for 1 h. DNA extraction was performed using the KingFisher Flex Purification system (Thermo Scientific, Singapore) using the NucleoMag Pathogen Kit (Macherey-Nagel GmbH, Germany) following the manufacturer’s instructions with a starting volume of 200 µL of bacterial suspension. The qPCR targeted the superoxide dismutase gene, using primers and probe sequences cited in Table 2. The reaction mixture (final volume 20 µL) contained 10 µL of Universal Master Mix (Applied Biosystems), primers at 0.8 µM, probe at 0.2 µM, and 5 µL of extracted DNA. Amplification was carried out on a Lightcycler 480 II (Roche Diagnostics) instrument as described previously (16).

**TABLE 2:** Probes and primers used for amplification of *C. burnetii*

Primer or probe	Sequence
Forward primer	CTTTTTACCGACTCCGCAAA
Reverse primer	ACGAGCGTTGACAGTGCTT
Probe	6-fam ATCATTTCGGCTCGGGATGGGC

## RESULTS

### Optimization of *C. burnetii* visualization using SEM and image generation

**Fig 3:**
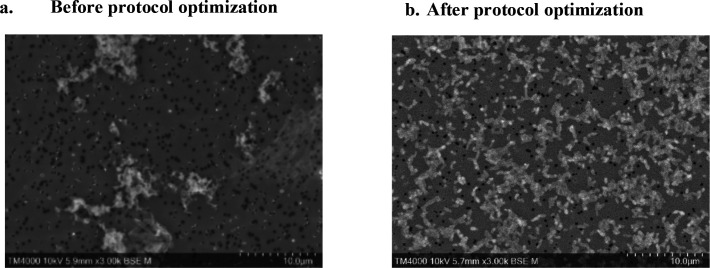
(**a**) SEM image of *C. burnetii* prior to protocol optimization. (**b**) SEM image *of C. burnetii* sample prepared with the optimized protocol.

The images taken before protocol optimization showed aggregated bacteria on the filter, and these images were not suitable for analysis (Fig. 3a). In contrast, images taken after protocol optimization demonstrated major improvement, highlighted by the reduced bacterial aggregation, which was evident after the addition of Tween 80. In fact, the latter reagent exerted no effect on bacterial morphology, as verified by SEM images for protocols with and without Tween 80. Other refinements included incorporating PTA as a contrasting agent and optimizing the wash step by using ACCM-2 medium. These refinements led to the optimized protocol that allowed the generation of high-quality SEM images (Fig. 3b). This refined protocol successfully eliminated bacterial aggregation, provided a homogenous bacterial distribution on the filter, and enhanced visualization. The turnaround time for the entire process was observed to be a mere 2 h, including the time needed for the inactivation of *C. burnetii*.

### Growth monitoring of *C. burnetii* strains

**Fig 4:**
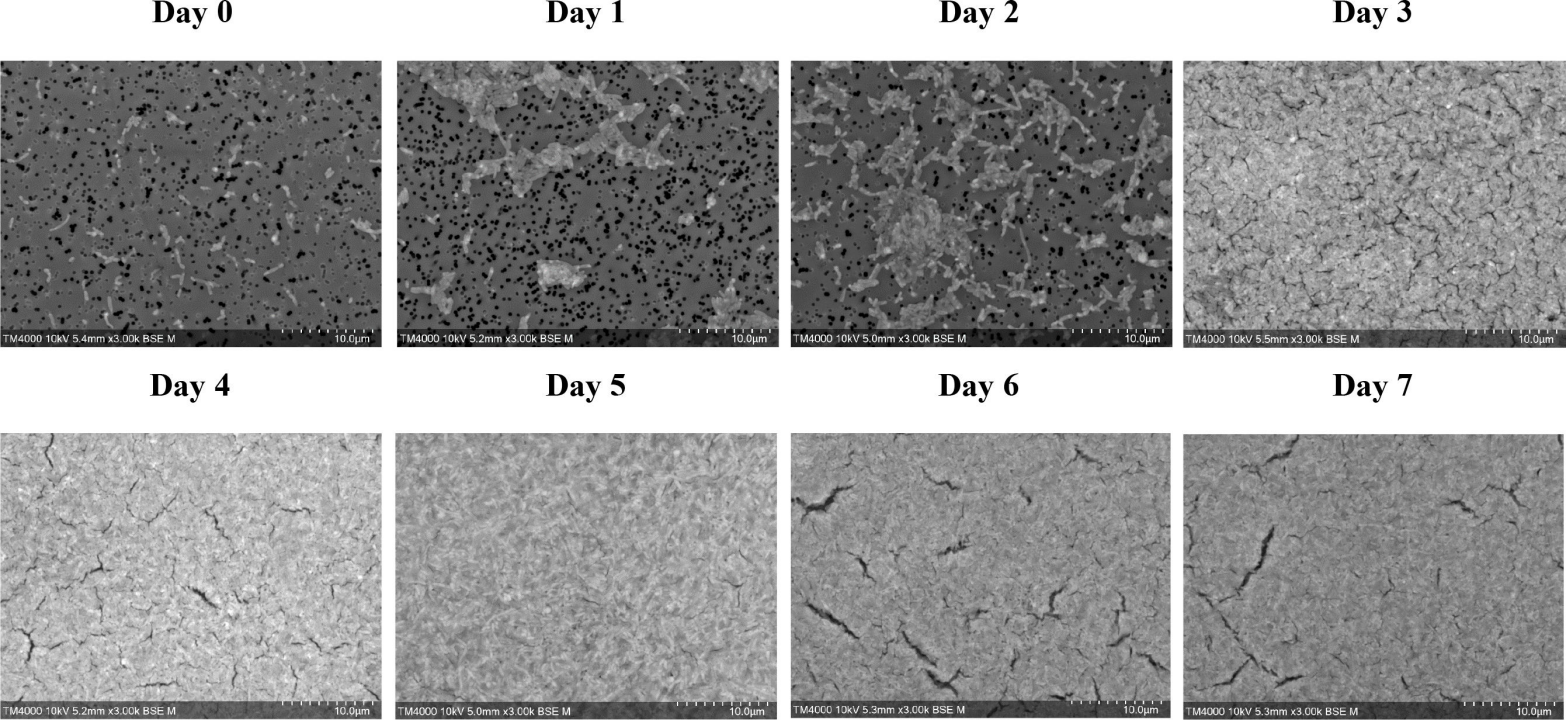
Growth monitoring and early detection of *Coxiella burnetii* strain 109.

The daily growth kinetics monitoring of *C. burnetii* over 1 week, following inoculation into axenic medium, using SEM, revealed a significant increase in bacterial density over time. Figure 4 shows the observed SEM images used for growth monitoring of the tested *C. burnetii* strains. The images showed a clear progression in bacterial density and distribution over time. Upon analyzing the results obtained, limited growth detection was observed as early as day 2. However, day 3 emerged as the first time point for which the bacterial population showed percentage area coverage >60% among all tested strains. This was further reflected in Table 3, where the growth on day 3 was found to be sufficiently dense with a percentage area coverage > 60%. Therefore, growth detection on day 3 using this innovative method is 70% earlier than that possible by traditional methods (minimum on day 7).

**TABLE 3:** Percentages of covered area calculated for eight *C. burnetii* strains

Time point	*C. burnetii* 336	*C. burnetii* Henzerling	*C. burnetii* 109	*C. burnetii* 346	*C. burnetii* 284	*C. burnetii* NMI	*C. burnetii* 252	*C. burnetii* 350
Day 0	2	6	6	5	7	7	10	5
Day 1	5	12	19	14	16	16	19	20
Day 2	20	27	23	26	32	46	50	40
Day 3	66	71	81	71	76	71	67	70
Day 4	70	78	85	76	78	75	69	70
Day 5	90	87	88	81	79	80	90	75
Day 6	92	91	92	93	90	93	93	77
Day 7	93	94	96	95	92	96	95	78

### Image analysis

**Fig 5:**
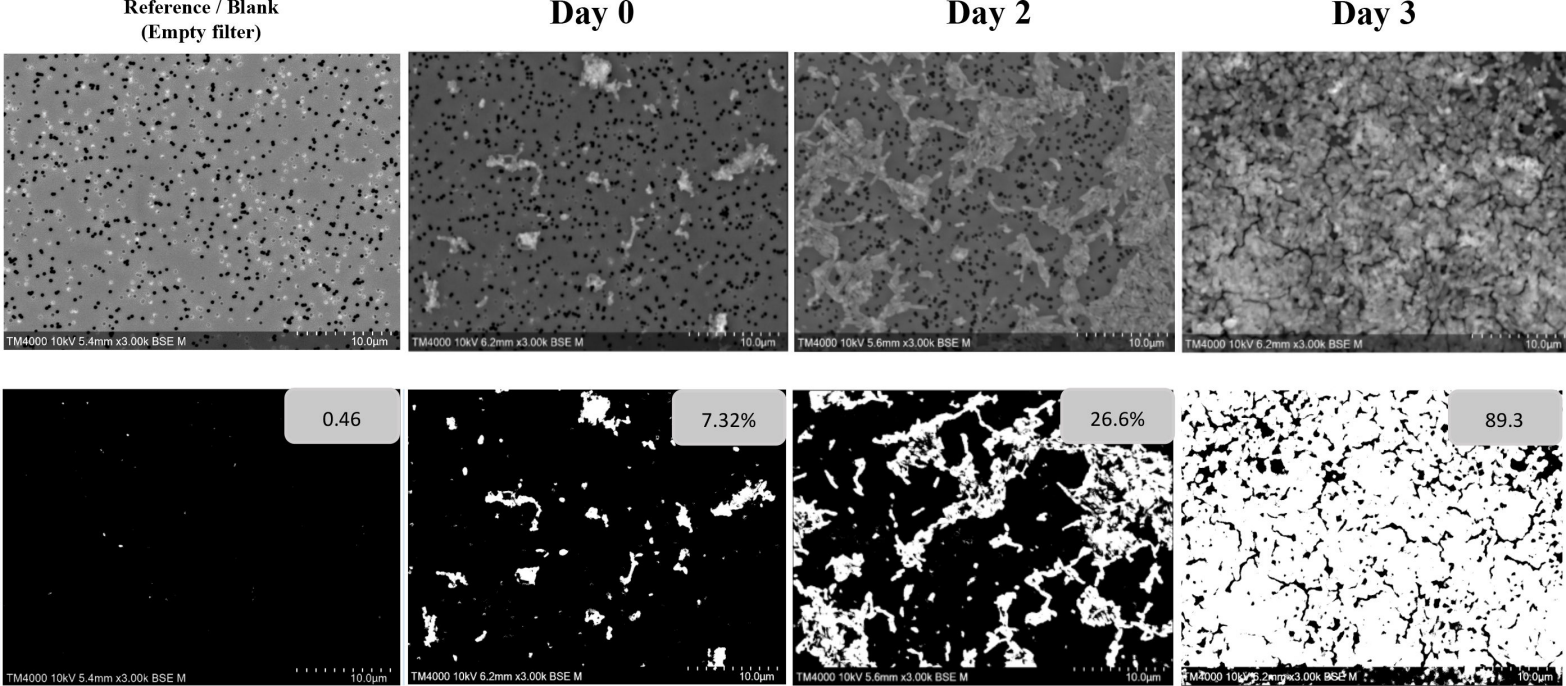
SEM-based growth monitoring comparing day 0 through day 3 to the reference/blank condition by image binarization with thresholding for percentage area coverage determination.

**Fig 6:**
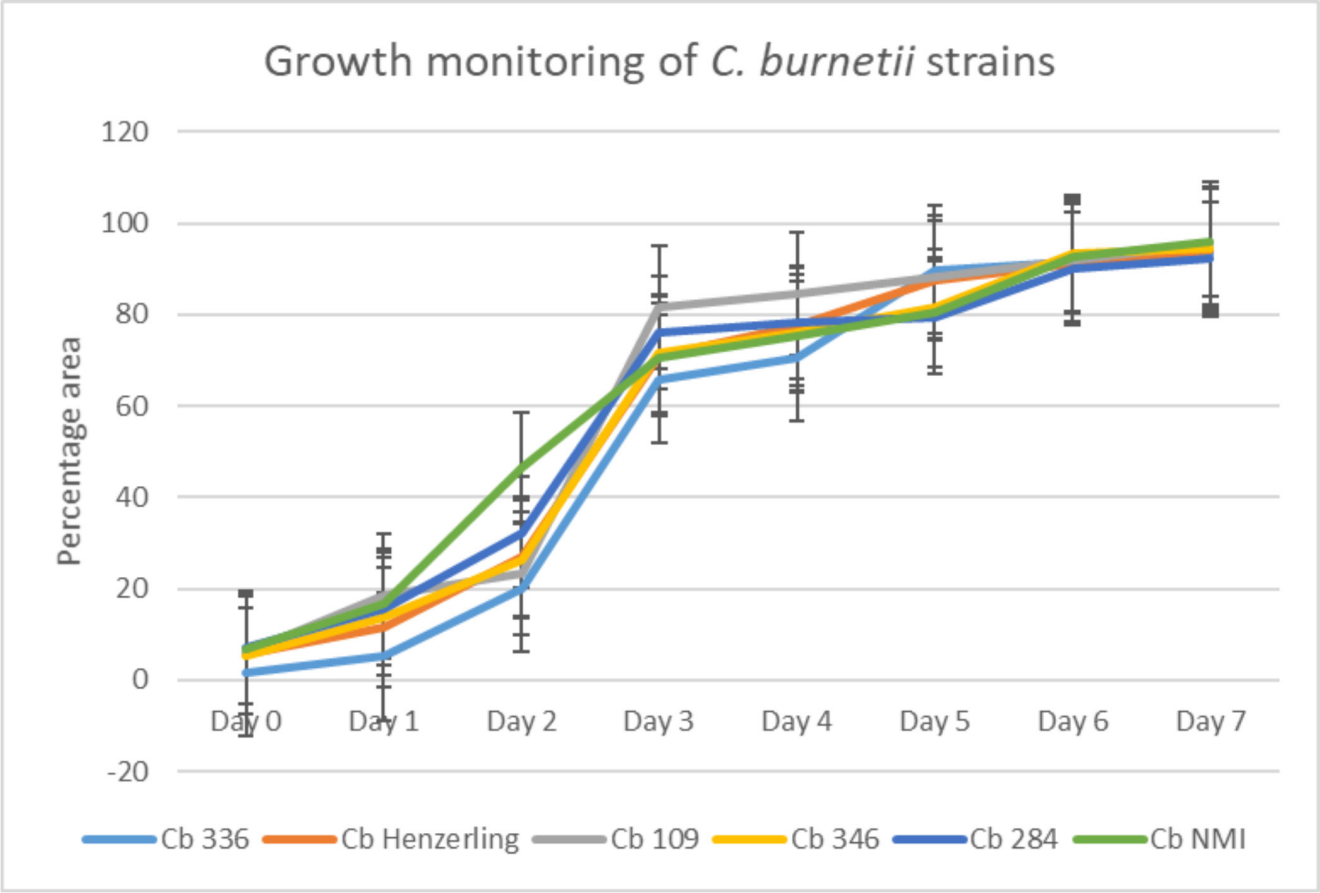
Growth monitoring of *C. burnetii* strains over 1 week.

Table 3 shows the chronological evolution of the percentage area covered by bacteria for all tested strains throughout the 7 days of observation. Figure 5 reveals an example of analyzed images used for SEM-based growth tracking, comparing the reference/blank to the observed culture conditions from day 0 to day 3. On day 0, the percentage area was less than 10% for all strains. By day 3, the percentage area significantly rose, reaching 60% among all strains. Percent area coverage measurements beyond day 3 confirmed continuous growth, however at a slower rate, with the percent area coverage reaching a plateau of 90–95% by day 7, as shown in Fig. 6.

### Bacterial growth detection by qPCR

The qPCR results were consistent with those obtained using our method. A Ct difference of approximately 3 was observed between day 0 and days 3–4, indicating the onset of bacterial multiplication from day 3 onwards and confirming active growth during the first week of culture in axenic medium (Table 4). These findings were comparable to those generated by our approach, thereby supporting the reliability and validity of our method. The early detection of growth further demonstrates the method’s effectiveness in monitoring the viability and proliferation of *Coxiella burnetii* in ACCM-2 medium.

**TABLE 4:** qPCR cycle threshold (Ct) values for *C. burnetii* strains

Days	Results of (Ct) CB Nine Mile	Results of (Ct) CB Henzerling	Results of (Ct) CB 346	Results of (Ct) CB 336
Day 0	19.80	20.58	20.28	20.27
Day 1	19.17	19.21	21.25	21.63
Day 2	17.06	17.19	18.51	18.32
Day 3	14.99	16.24	16.24	18.03
Day 4	13.03	14.74	13.23	15.66
Day 5	11.48	13.26	12.22	15.49
Day 6	10.95	12.02	11.94	15.13
Day 7	10.54	11.23	11.45	14.76

### Statistical analysis

No significant difference (*P* value < 0.05) was detected when comparing the percentage area coverage of each day among the eight strains. Thus, the early detection using SEM was reproducible among the eight studied strains. In addition, the time point “day 3” with a threshold of 60% covered area was found among all studied strains.

## DISCUSSION

The results of this study highlight the utility of SEM) for the early detection of *C. burnetii* growth in axenic medium. The rapid detection of bacterial growth is a persistent challenge in microbiology, especially for fastidious pathogens like *C. burnetii*. These challenges highlight the need for the development of an innovative approach that can accelerate detection while providing reliable insights into microbial growth. Early growth detection remains crucial in cases of outbreaks, emerging acquired resistance, and therapeutic failure, especially in chronic infection. This would enable more effective interventions, improvement of patient outcomes, and better control of outbreaks. Moreover, the development of innovative early detection techniques is required to advance research and open new perspectives for understanding this pathogen.

Our study presents a novel approach and demonstrates a proof-of-concept for an early detection of *C. burnetii* growth using a tabletop SEM coupled with axenic culture medium (ACCM-2) by using a rapid and simple method to prepare the samples.

This approach enabled the detection of significant growth of *C. burnetii* within 3 days in comparison with conventional methods that are complex and time-consuming, typically requiring 2–3 weeks (17). Notably, the work by Francis et al*.* using immunofluorescence for growth tracking required 10 days for accurate detection of bacterial growth and 15 days for performing cell culture (7). Immunofluorescence techniques depend on the availability of specific antibodies and are unable to assess cell viability (18). Moreover, culture on solid axenic agar confirms bacterial viability through colony formation but is slow and can take between 15 and 21 days for the colonies to grow on solid media (19). Concerning molecular techniques such as qPCR, they offer a rapid and sensitive alternative by detecting *C. burnetii* through bacterial DNA (18). However, a key limitation of qPCR is its inability to discriminate between DNA from live and dead cells, which may lead to overestimation of viable pathogens in a sample. On the other hand, our approach consists of a rapid protocol that enables SEM observation and growth detection faster and simpler than traditional methods commonly used for monitoring *C. burnetii* growth. Besides the simplicity and the rapidity, this approach provides complementary information by allowing direct visualization of bacterial morphology by preserving the bacterial structure. This structural preservation allows for a more comprehensive understanding of bacterial physiology and viability, replication dynamics and mechanisms of persistence, through morphological changes and staining with viability markers such as PTA (Sigma-Aldrich) which molecular DNA-based assays alone cannot provide. In addition, the turnaround time for SEM observation is 1 h 50 min including 1 h of deactivation compared with a turnaround of almost 3 h for the qPCR. With SEM, we were able to observe significant bacterial growth as early as day 3, a finding not previously described using qPCR (20). Table 5 represents a comparison of different methods used for growth monitoring. Another major advantage for using SEM was the accurate observation of bacterial morphology and growth starting the first days of cultivation, an aspect that was inaccessible with other techniques. This also allowed for early detection of possible culture contamination. Furthermore, observing bacterial morphology from day 0 provided insight into the viability status of the cultured bacteria, preventing unnecessary prolonged culture time for visibly deformed bacteria that may be non-viable.

**TABLE 5:** Comparison of methods for detection and viability assessment of *C. burnetii*

Method	Advantages	Turnaround time	Time for first growth detection	Disadvantages
SEM + ACCM-2 medium	✓ Direct morphological detection of bacteria ✓ Early visualization of growth ✓ Potential indication of viability via morphological changes and staining	1 h 40 min (including deactivation time)	3 days	The fixation protocol to be optimized in order to stain live and dead cells and distinguish between them.
qPCR + ACCM-2 medium	✓ Rapid ✓ Highly sensitive ✓ Quantitative detection of bacterial DNA	3 h	3–4 days	Does not differentiate live vs. dead cells No morphological information provided
Culture on solid axenic agar (ACCM-2 agar)	✓ Detection of viable bacteria by colony growth	Less than 1 h	7–21 days	Slow Difficult to grow colonies for *C. burnetii* on solid medium Labor-intensive
Cell culture + immunofluorescence	✓ Specific detection via antibody fluorescent labeling	2 h	~10 days	Requires availability of specific antibodies Does not distinguish cell viability

From a technical point of view, the strength of this innovative tabletop SEM technology lies in its capacity to rapidly generate high-resolution images under low vacuum conditions. Moreover, it is user-friendly and does not require extensive expertise for operation, making it accessible to a wider range of researchers, thereby overcoming common limitations of traditional SEM such as sample charging and image degradation during extended or repeated observations. This novel approach provides quantitative data through percentage area coverage by using ImageJ Fiji Software, confirming significant growth by day 3. These findings have important implications for research. Concerning the application of these techniques on clinical samples, Boden et al. (15) previously demonstrated the feasibility of isolating *C. burnetii* from clinical specimens using ACCM-2 (15). Therefore, combining this approach in the future with direct culture of clinical samples in axenic medium could present an important step forward, potentially enhancing the efficiency of Q fever diagnosis and advancing the understanding of *C. burnetii* growth dynamics in axenic conditions (21).

Nevertheless, this study has certain limitations, including a small sample size. Further research will expand the number of tested strains to provide more information on the reproducibility and stability of this approach. Furthermore, the current image analysis strategy can benefit from a semi-automated or automated application, ideally coupling automated imaging with real-time analysis. Recent advances in machine learning and artificial intelligence integration in diagnostic tools could eventually propel this method forward toward larger-scale applications of clinical and scientific interest.

On the other hand, *C. burnetii* remains a Category 3 microorganism that can only be manipulated in a BSL3 laboratory. This prevents the observation of samples without complete inactivation of the pathogen, which involves prolonged exposure to fixatives that can sometimes alter bacterial membranes and surface structures. Addressing this challenge possibly involves the integration of novel tabletop SEMs in BSL3 settings, facilitating the observation of samples following simple fixation rather than complete inactivation. At that point, further SEM-based analyses including bacterial viability determination using PTA (Sigma-Aldrich).

Given these findings, the combination of SEM with axenic culture and molecular assays represents a robust approach for rapid, reliable, and phenotypically informative *C. burnetii* detection, which may enhance real-time outbreak management and epidemiological surveillance. Finally, this early growth detection approach for *C. burnetii* opens the door for wider research applications such as optimization of axenic culture of *C. burnetii*, antimicrobial susceptibility testing, and rapid isolation of the bacterium in epidemic situations, including the analysis of environmental samples and effective outbreak investigation.

## Data Availability

The original contributions presented in the study are included in the article and supplemental material. Further inquiries can be directed to the corresponding author.

## SUPPLEMENTAL MATERIAL

Fig. S1 spectrum.01011-25-s0001.pdf Electron microscopy images of a representative strain.

ASM does not own the copyrights to Supplemental Material that may be linked to, or accessed through, an article. The authors have granted ASM a non-exclusive, world-wide license to publish the Supplemental Material files. Please contact the corresponding author directly for reuse.
