# Supplementary figures and images for: Early detection of Coxiella burnetii growth on axenic media using scanning electron microscopy
Source: Microbiol Spectr. 2025 Dec 23;14(2):e01011-25. doi: 10.1128/spectrum.01011-25 (PMC12889089; doi:10.1128/spectrum.01011-25)

## Supplementary Data

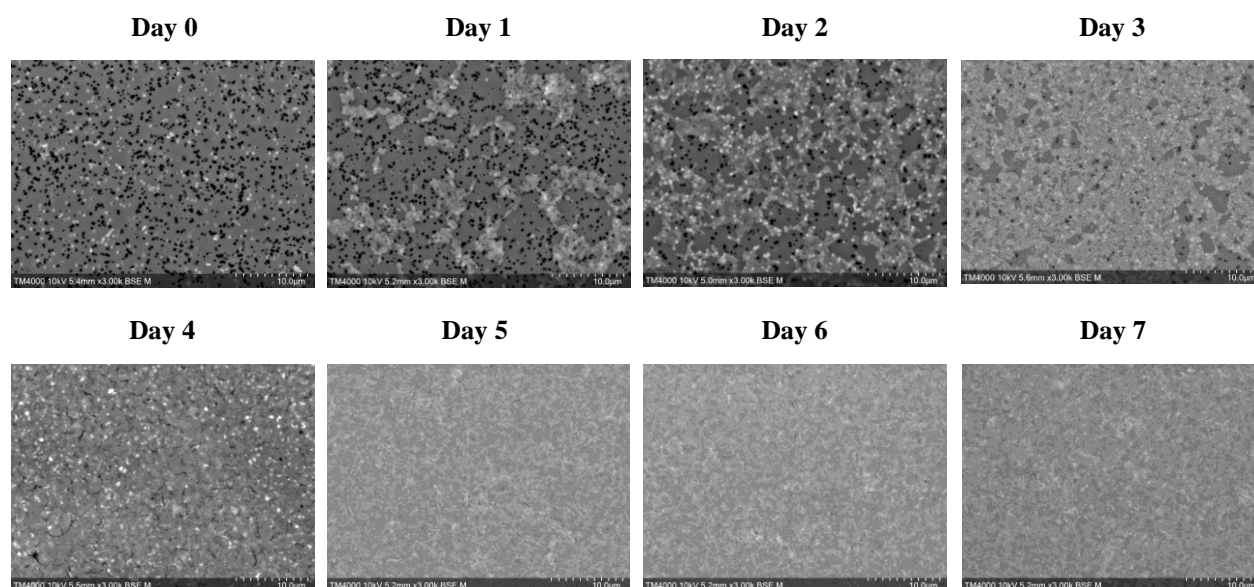

Supplement: Fig. S1 — Electron microscopy images of a representative strain. [file spectrum.01011-25-s0001.pdf]
